# An integrated multicriteria decision making framework for the selection of waste cement dust filled automotive brake friction composites

**DOI:** 10.1038/s41598-023-46385-5

**Published:** 2024-03-21

**Authors:** Tej Singh

**Affiliations:** Savaria Institute of Technology, Faculty of Informatics, ELTE Eötvös Loránd University, Szombathely, 9700 Hungary

**Keywords:** Engineering, Materials science, Mathematics and computing

## Abstract

This work discusses selecting optimal brake friction composite alternatives based on an integrated MABAC (multi-attributive border approximation area comparison) and AHP (analytic hierarchy process) approach. Therefore, non-asbestos automotive brake friction composites containing varying proportions of cement dust (50 to 0 wt%) and barium sulfate (0 to 50 wt%) were developed and tribo-evaluated on a Krauss machine following European regulations. Composite made up of 30 wt% cement dust and 20 wt% barium sulfate had the highest friction coefficient (0.361), lowest variability coefficient (0.598), and maximum recovery (123.27%). The composite with the least fading (15.36%) included 50 wt% cement dust, whereas the composite with the lowest wear (9.10 g) and the least frictional fluctuations (0.271) contained 50 wt% barium sulfate. By AHP, the friction coefficient (0.1989), fade (0.1696), recovery (0.1551), and wear (0.1412) were selected as the essential criteria in the performance assessment. Based on the MABAC ranking evaluation, the composite comprises 20 wt% barium sulfate and 30 wt% cement dust has the best tribological profile, whereas the composites of solely cement dust or barium sulfate have the poorest tribological profile. The acquired ranking results were confirmed using other decision-making models and subjected to sensitivity analysis to demonstrate their robustness.

## Introduction

Nowadays, more emphasis is being placed on environmental consciousness and the importance of a circular economy. Unmanaged waste causes environmental issues, while its managed use can promote a circular economy^1^. Any society's advancement is mainly dependent on the availability of high-quality goods. Industries play a crucial role in transforming basic materials into valuable goods. Any industrial activity generates various kinds of waste^2^. Unregulated waste disposal harms our health and the environment^3^. In addition, the term "circular economy" is used to characterize a zero-waste strategy in which waste from one product is used as raw material for making a completely different product^4^. This method will help reduce reliance on traditional materials and assist in environmental mitigation. Several studies have recently been conducted to evaluate the potential of waste materials for diverse uses and be better than the regularly used resources^5–8^. One such application is automotive brake friction materials, where waste materials might be used to cut production costs and control the health risks associated with waste disposal^9,10^.

Automotive friction materials must be multi-component composites that produce the required tribological properties during braking. These composites include over twenty materials classified as reinforcing fibers, polymers as binders, fillers, abrasives, and lubricants^11^. Hybrid composites with filler contents between 30 and 70 wt% are a good fit for an economy-driven approach to friction materials for automobiles made from waste^12^. Dadkar et al.^13^ looked at the tribological properties of a mixture of flyash (65–80 wt%) and phenolic resin (20–35 wt%). According to the authors, the composites with the highest flyash concentration (80 wt%) had the most significant values of friction and recovery with the slightest fading. Singh et al.^14,15^ determined that the size of cement dust and its compatibility are critical in influencing the tribological performance of composites. Wang et al.^16^ showed that adding slag waste to friction composites enhanced thermal, mechanical, and tribological properties. The influence of flyash and zinc borate combinations on the tribological properties of automobile brake composites was examined by Öztürk and Mutlu^17^. The authors stated that composites containing 60–65 wt% flyash and 0–5 wt% zinc borate had better fade resistance and friction stability. However, the wear of the composites deteriorated with increasing flyash content and improved with decreasing zinc borate content. Ahlawat et al.^18^ studied the tribological behavior of a friction composite made from milled and raw flyash. According to the authors, including 35 wt% of milled raw flyash resulted in the highest coefficient of friction but at the cost of increased wear. Jayashree and Straffelini^19^ investigated the influence of recycled aluminum anodizing waste on phenolic-based brake friction materials. Consistent addition of anodizing waste increased the coefficient of friction of composites but at the expense of wear resistance. Ding et al.^20^ investigated the utilization of recycled polyimide powder in friction material applications. The authors claim that composites' fade resistance and friction stability has been enhanced at elevated temperatures. Binda et al.^21^ examined the use of waste slate powder in automobile brake friction composite materials. The results demonstrated that as the slate powder concentration increased, the friction coefficient remained nearly constant while composite wear increased. In recent studies, Jayashree et al.^22,23^ investigated the potential of slag waste in automotive braking applications. Compared to the reference composition, slag-loaded formulations exhibit higher friction performance with almost the same wear performance and particulate emission.

The tribological characteristics of automotive brake friction composites are determined mainly by the type and amount of ingredients used^24^. Therefore, formulation design is an issue that material scientists often face. Moreover, formulation designers have to deal with the problem concerning several conflicting evaluation criteria, like the higher coefficient of friction and recovery performance with lower wear and fade performance, which make the formulation design more complicated^25^. The multi-criteria decision-making (MCDM) is an excellent answer for this situation^26^. Several MCDM models have been reported in recent years to pick the best formulation^25,27–29^. The AHP (analytic hierarchy process) and MABAC (multi-attributive border approximation area comparison) are popular and extensively applied to solve decision-making problems^30–32^. The AHP discusses using a pairwise comparison matrix to calculate the weight of criteria. In contrast, the MABAC technique analyzes the ranking of alternatives based on their distances from the border approximate area solution. For optimal solution, the hybrid AHP-MABAC approach was prosperously utilized for electric vehicle selection by Sonar et al.^32^, for geometric and flow parameters optimization in solar air collectors by Salman et al.^33^, in sustainable building construction by Soni et al.^34^ and for flood simulation models by Tabarestani and Afzalimehr^35^.

Therefore, in an earlier study, automotive brake friction composites filled with cement dust and barium sulfate fabricated and characterized for tribological properties have been used in the present research work^36^. Due to the difficulty in selecting the best-manufactured automotive brake friction composites, the hybrid AHP-MABAC approach has been proposed. The assessed tribological properties are fixed as the selection criteria, AHP is used to calculate the criterion weight, and the MABAC technique is used to derive the final composite ranking. This study presents a novel approach to addressing the issue of waste generated by the cement industry in the development of automotive brake friction materials. The suggested approach involves using waste cement dust in its original form, without undergoing any modifications, to develop braking materials. This approach will not only aid in mitigating environmental issues related to waste disposal but will also concurrently diminish our reliance on conventionally utilized materials. This research discusses using waste cement dust to develop automotive brake friction materials, making them efficient, cost-effective, and environmentally sustainable. The following is a summary of the study's key contributions:

- This work makes a valuable contribution to the design and development of waste cement dust-filled sustainable composite materials tailored explicitly for use in automotive braking applications.
- The issue concerning the selection of composite materials has been effectively addressed via an integrated AHP-MABAC-based MCDM technique.
- In order to assess the consistency and robustness of the proposed integrated MCDM technique, a sensitivity analysis has been conducted.
- The comparative analysis has been conducted to evaluate the ranking outcomes of the suggested alternatives for composite materials selection concerning other existing MCDM techniques.

The following is the outline of the study. Section “Experimental work” presents the experimental work involving the fabrication of composites, the selection of criteria, and the use of integrated optimization techniques. The findings and analysis, both experimental and mathematical, are presented in Section “Results and discussion”. The conclusions are drawn in Section “Conclusions”.

## Experimental work

### Formulation detail

**Figure 1:**
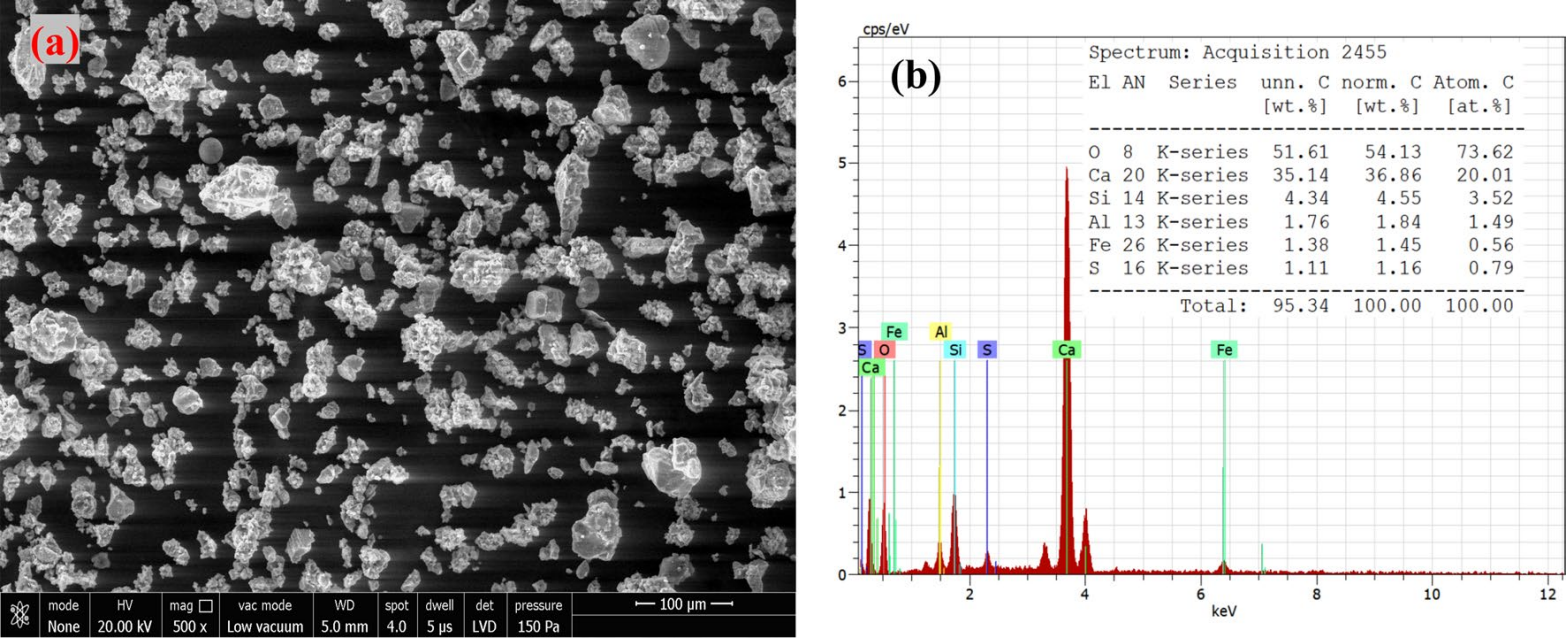
(**a**) SEM and (**b**) EDS analysis of used cement dust.

The formulations are designed on a fixed composition (50 wt%) which includes binder (phenolic resin), fibers (Kevlar, steel, lapinus), property modifiers (alumina, vermiculite, graphite). Cement dust (325 mesh; 50 to 0 wt%) and barium sulfate (325 mesh; 0 to 50 wt%) were used to adjust the remaining 50 wt%, as shown in Table 1. Cement dust was obtained from one of the cement industries in Himachal Pradesh, India. Figure 1 shows the scanning electron microscope image (SEM) and energy-dispersive spectroscopy (EDS) spectrum of utilized cement waste. The EDS verifies the presence of Si (primarily for SiO_2_), Ca (primarily for CaO), Al (mainly for Al_2_O_3_), S (mainly for SO_3_), and Fe (mainly for Fe_2_O_3_)^37^.

**Table 1:** Nomenclature and compositional detail.

Ingredients (wt%)	Dimensions	Nomenclature						
		CD1	CD2	CD3	CD4	CD5	CD6	CD7
Phenolic resin	200 mesh	10	10	10	10	10	10	10
Lapinus fiber	Length = 100–150 μm; Diameter = 5 μm	10	10	10	10	10	10	10
Steel fiber	Length = 1–2 mm; Diameter = 150–200 μm	10	10	10	10	10	10	10
Kevlar fiber	Length = 12 μm; Diameter = 1–2 mm;	5	5	5	5	5	5	5
Alumina	200 mesh	5	5	5	5	5	5	5
Graphite	325 mesh	5	5	5	5	5	5	5
Vermiculite	0.5–1 mm	5	5	5	5	5	5	5
Cement dust	325 mesh	50	40	30	25	20	10	0
Barium sulfate	325 mesh	0	10	20	25	30	40	50

### Fabrication details

Materials were weighed and mixed for 10 min in a 3000 rev/m shear mixer for composite formation. To make brake pads preform, 72 g of each formulation was mixed and pressed in a hydraulic cold press at 90 kg/cm^2^. After that, the preform is heated to 155 °C and subjected to a constant pressure of 10 MPa for ten minutes while adhesive-coated steel backplates are used in molding. In order to alleviate the generated residual stresses and prevent the void formation in the composites, five intermittent breaths were administered at various times throughout the temperature molding process. After being molded, the composites underwent a post-curing process that involved heating them in an oven at 170 °C for four hours. Finally, the brake composites were machined to the desired thickness before being used for tribological measurements.

### Tribological testing and selection of criteria

Under European standards, in particular, ECE (Economic Commission for Europe) Regulation (R) 90, a computer-controlled Krauss machine was utilized to conduct a tribological evaluation of the composites produced. The detailed operation of the machine and the ECE R90 protocol are extensively documented in the literature^36^. The procedure includes 100 brakings, with 30 brakings for bedding and 70 for the primary assessment. The 30 bedding brakes were used to ensure that the brake composite and revolving disc were in uniform contact. With the help of cooling, the bedding was started at 100 to 280 °C. After 30 bedding brakes, the primary performance test was started, which consisted of seven 10-brake cycles (one for cold, five for fade, and one for recovery), as detailed in Table 2^36^.

**Table 2:** Experimental conditions.

	Bedding cycle	Cold cycle	Fade cycle	Recovery cycle
Number of brakes	30	10	50	10
Speed (rpm)	660	660	660	660
Initial temperature (°C)	100	45	100	100
Braking pressure (N/cm^2^)	200	200	200	200
Cooling	On	On	Off	On

The testing findings were analyzed in friction performance, stability coefficient, fade (%), friction fluctuations, recovery (%), variability coefficient, and maximum rise in disc temperature and used as selection criteria. Additionally, it is necessary to investigate other parameters related to the efficacy of the developed friction materials, including factors such as the release of airborne particles and the occurrence of brake squeal noise, which can be investigated in future research efforts^38,39^. Table 3 contains a complete overview of the selected criteria and their performance implications^36^.

**Table 3:** Selected criteria.

Criteria	Performance indication	Description
$C1$: µ_P_, performance coefficient of friction	Higher is good	Calculated as the average of the μ recorded for the 70 primary braking instances
$C2$: wear (g)	Lower is good	Computed by measuring the weight loss of sample before and after test
$C3$: fade (%)	Lower is good	Determine as, fade (%) = $\frac{{{\upmu }_{{\text{P}}} - {\upmu }{}_{fade}}}{{{\upmu }_{{\text{P}}} }} \times 100$, where ${\upmu }{}_{fade}$ is the lowest attained friction coefficient value for fade cycles taken after 270 °C
$C4$: recovery (%)	Higher is good	Determine as, recovery (%) = $\frac{{{\upmu }_{{{\text{recovery}}}} }}{{\upmu }} \times 100$, where, ${\upmu }_{{{\text{recovery}}}}$ is the highest attained friction coefficient value for the recovery cycle
$C5$: stability coefficient	Higher is good	Determine as, stability coefficient = $\frac{{{\upmu }_{{\text{P}}} }}{{{\upmu }_{\max } }}$, where ${\upmu }_{\max }$ is the highest friction coefficient value for the 70 primary braking instances
$C6$: variability coefficient	Lower is good	Determine as, variability coefficient = $\frac{{{\upmu }_{\max } - {\upmu }_{{{\text{min}}}} }}{{{\upmu }_{\max } }}$, where, µ_min_ is the minimum friction coefficient value for the 70 primary braking instances
$C7$: friction fluctuations	Lower is good	It is calculated as the difference of µ_max_ and µ_min_
$C8$: maximum disc temperature rise (^o^C)	Lower is good	It is taken as the maximum disc temperature rise (DTR_max_) for the test

### Optimization methodology

**Figure 2:**
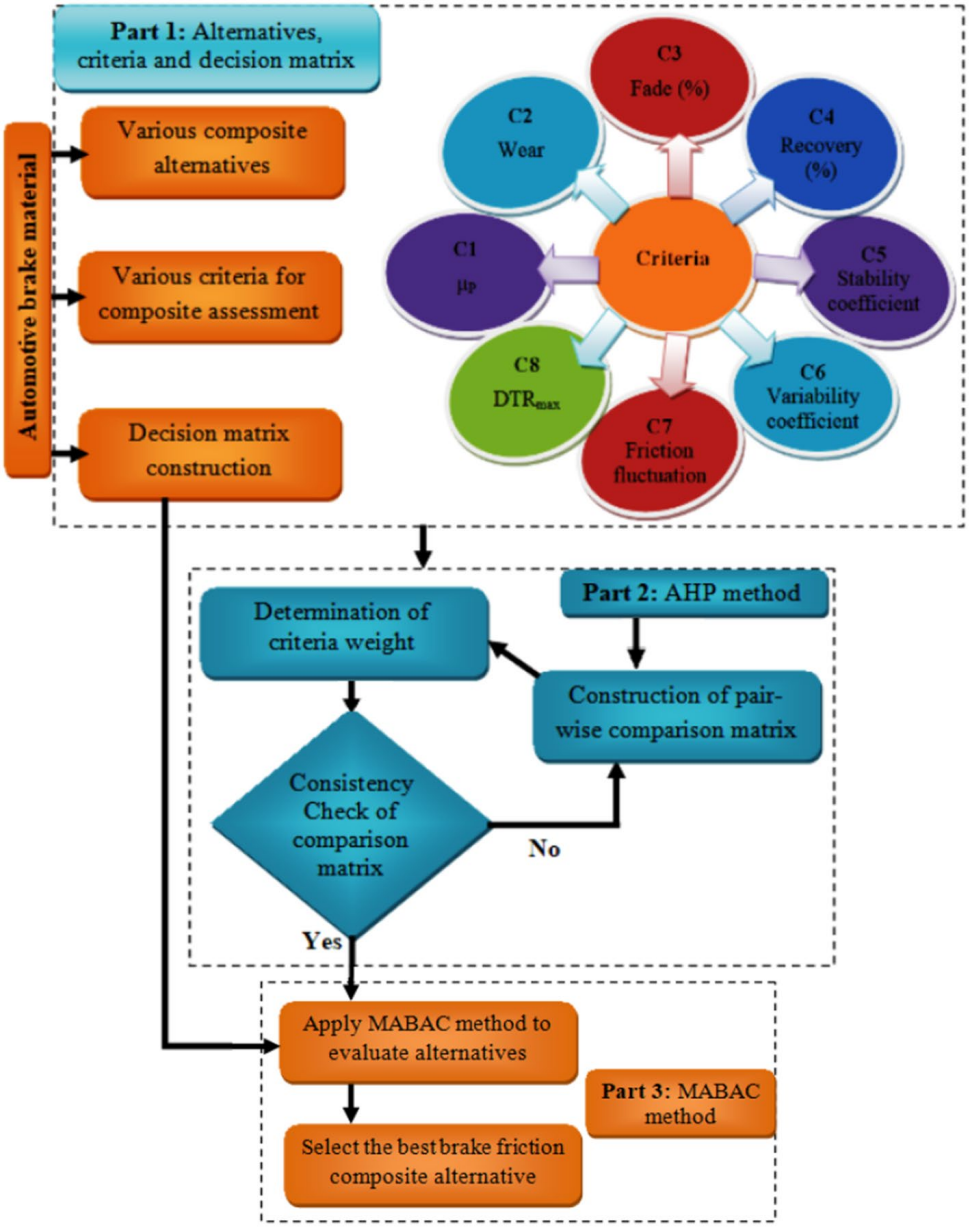
The overview of the proposed algorithm.

This work proposes an integrated AHP-MABAC technique for the performance assessment and ranking of the best waste cement dust-filled friction composite material alternatives. The study uses the AHP to determine the criterion weight and then utilizes the MABAC technique to identify the optimal composite alternative. One notable benefit of the MABAC technique is its applicability to addressing complex situations that include several criteria and alternatives. This benefit arises from the method's capacity to manage the mathematical formulation of the issue without becoming too complicated, even as the number of criteria and alternatives increases^40,41^. Furthermore, the MABAC approach provides reliable responses in situations when there is a shift from a favoured criterion type to a less favoured criterion type^40^. The use of the AHP approach offers notable benefits in comparison to other weighting methodologies. The AHP approach facilitates pairwise comparisons, which enhance the precision of assessments in contrast to the simultaneous evaluation of all possibilities. Additionally, this approach addresses both physical and intangible characteristics. Furthermore, by including a consistency check, decision-makers can verify the accuracy of their assessments^42^. The AHP weighting approach is advantageous as it allows for the inclusion of valuable perspectives from experienced decision-makers. Nevertheless, it is essential to recognize that these observations may sometimes demonstrate a predisposition towards specific criteria due to the decision-makers pre-existing opinions. Consequently, this prejudice may lead to results that are influenced by bias. Furthermore, the level of complexity associated with this approach increases as the number of factors involved in the decision-making challenge expands^42^. The proposed AHP-MABAC technique contains three main stages of assessment process as presented in Fig. 2.

#### Stage 1: determination of alternatives, criteria and decision matrix

In first stage the alternatives and criteria of a given MCDM problems are specified and arranged in the form of a decision matrix. For *p* alternatives $\left( {x_{i} ,\;i = 1,\,\,2, \cdots ,\,\,p} \right)$ and* q* criteria $\left( {C_{j} ,\;j = 1,\,\,2, \cdots ,\,\,q} \right)$ the decision matrix can be formulated as:

1 $$\Gamma_{p \times q} = \begin{array}{*{20}c} {x_{1} } \\ {x_{2} } \\ \begin{gathered} \vdots \hfill \\ x_{i} \hfill \\ \vdots \hfill \\ \end{gathered} \\ {x_{p} } \\ \end{array} \mathop {\left| {\begin{array}{*{20}c} {\Gamma_{11} } & {\Gamma_{12} } & \ldots & {\Gamma_{1j} } & \ldots & {\Gamma_{1q} } \\ {\Gamma_{21} } & {\Gamma_{22} } & \ldots & {\Gamma_{2j} } & \ldots & {\Gamma_{2q} } \\ \vdots & \vdots & \ldots & \vdots & \ldots & \vdots \\ {\Gamma_{i1} } & {\Gamma_{i2} } & \ldots & {\Gamma_{ij} } & \ldots & {\Gamma_{iq} } \\ \vdots & \vdots & \ldots & \vdots & \ldots & \vdots \\ {\Gamma_{p1} } & {\Gamma_{p2} } & \ldots & {\Gamma_{pj} } & \ldots & {\Gamma_{pq} } \\ \end{array} } \right|}\limits^{{\begin{array}{*{20}c} {C_{1}} & {C_{2}} & {\cdots } & {C_{j} } & {\cdots } & {C_{q} } \\ \end{array} }}$$

The $\Gamma_{ij}$ value in the in the constructed decision matrix ($\Gamma_{p \times q}$) represent the experimental results of the $i{\text{th}}$ alternative, $x_{i}$, with respect to the $j{\text{th}}$ criterion, $C_{j}$.

#### Stage 2: AHP method for weight calculation

Saaty is credited with developing AHP, which is now a popular MCDM application. The AHP method involves the construction of a pairwise comparison matrix in order to arrive at an estimate of the relative significance of the criteria that are involved in a decision-making problem^30,43^. For *q* criteria, $\left( {C_{j} ,\;j = 1,\,\,2, \cdots ,\,\,q} \right)$ the formulated pairwise comparison matrix can be represented as:

2 $${\rm A}_{qq} = \begin{array}{*{20}c} {C_{1} } \\ {C_{2} } \\ \vdots \\ {C_{q} } \\ \end{array} \mathop {\left[ {\begin{array}{*{20}c} {{\rm A}_{11} }{{\rm A}_{12} }{{\rm A}_{13} \cdots }{{\rm A}_{1q} } \\ {{\rm A}_{21} }{{\rm A}_{22} }{{\rm A}_{23} \cdots }{{\rm A}_{2q} } \\ \vdots \vdots \vdots \vdots \\ {{\rm A}_{q1} }{{\rm A}_{q2} }{{\rm A}_{q3} \cdots }{{\rm A}_{qq} } \\ \end{array} } \right]}\limits^{{\begin{array}{*{20}c} {C_{1} \;\;\;} & {C_{2} \;\;} & {C_{3} \; \cdots } & {C_{q} } \\ \end{array} }} \quad {\rm A}_{ij} = 1\;{\text{if}}\;i = j,\;{\rm A}_{ji} = \frac{1}{{{\rm A}_{ij} }},\,{\rm A}_{ij} \ne 0$$

To formulate the comparison matrix ${\rm A}_{qq}$, the designers make pairwise comparisons between the different tribological properties taking part in the decision. The developed pairwise comparison matrix is based on the standardized comparison scale of nine levels. The pairwise comparisons are made to determine which element is more dominant. The following relationship can be used to determine the number of comparisons.

3 $${\text{Number}}\;{\text{ of }}\;{\text{comparisons }} = \frac{{q\left( {q - 1} \right)}}{2}$$

Normalization of the geometric mean method is used to determine the weight ($\omega_{i}$) of each criteria as;

4 $$\omega_{i} = \frac{{\left\{ {\prod\nolimits_{j = 1}^{q} {{\rm A}_{ij} } } \right\}^{\frac{1}{q}} }}{{\sum\nolimits_{i = 1}^{q} {\left\{ {\prod\nolimits_{j = 1}^{q} {{\rm A}_{ij} } } \right\}^{\frac{1}{q}} } }}\;\quad i = 1,\,2, \ldots ,\,q$$

A consistency ratio (CR) is calculated to find inconsistencies in the evaluation. It assesses the assessments' consistency compared to large samples of completely random judgments. The CR can be estimated as shown in the following expression.

5 $$CR = \frac{{\lambda_{\max } - q}}{q - 1} \times \frac{1}{RI}$$

Here, $\lambda_{\max }$ is the largest eigenvalue determined by applying the method outlined in reference^32^. The value of the random consistency index (RI) was chosen in accordance with the criteria^30–32^. The maximum CR number that can be used with the AHP methodology is 10%. The evaluation must be repeated if the final CR value exceeds 10% to ensure consistency^44^.

#### Stage 3: MABAC method for final ranking

MABAC is a dependable and widely-applied MCDM technique that identifies the best candidate alternative based on its proximity to the border approximation area solution^32,33,41^. In Eq. (1), the experimental results are shown as a decision matrix that is used to analyze the MABAC method. Following are the different steps to solve MABAC:

Step I: After constructing the decision matrix [Eq. (1)], normalization is performed concerning the selected criteria's desired preference implication. The normalized decision matrix ($\Re_{ij}$) was formulated using the following equations.

For preferable (higher is better) criteria:

6 $$\Re_{ij} = \frac{{\Gamma_{ij} - \Gamma_{i}^{ - } }}{{\Gamma_{i}^{ + } - \Gamma_{i}^{ - } }}$$

For non-preferable (lower is better) criteria:

7 $$\Re_{ij} = \frac{{\Gamma_{ij} - \Gamma_{i}^{ + } }}{{\Gamma_{i}^{ - } - \Gamma_{i}^{ + } }}$$

where, $$\Gamma_{i}^{ + } = \max (\Gamma_{1} ,\;\Gamma_{2} ,\;\Gamma_{3} , \ldots \Gamma_{p} )$$, and $$\Gamma_{i}^{ - } = \min (\Gamma_{1} ,\;\Gamma_{2} ,\;\Gamma_{3} , \ldots \Gamma_{p} )$$.

Step 2: After that, elements of weighted normalized decision matrix are calculated as:

8 $$\Re_{ij}^{ * } = \omega_{j} + (\Re_{ij} \times \omega_{j} )$$

Step 3: In this step border approximation area matrix is constructed as:

9 $$\eta_{j} = \left( {\prod\limits_{i = 1}^{p} {\Re_{ij}^{ * } } } \right)^{1/p}$$

Step 4: The distance of elements in weighted normalized decision matrix is determined from the formulated border approximation area matrix as:

10 $$\delta_{ij} = \Re_{ij}^{ * } - \eta_{j}$$

Step 5: The overall score index of each alternative is computed using following equation.

11 $$\Phi_{i} = \sum\limits_{j = 1}^{q} {\delta_{ij} }$$

Step 6: The $\Phi_{i}$ number was used, in the end, as the basis for the ranking decision. The alternative that has the greatest $\Phi_{i}$ value is presented first in the listing, while the alternative that has the smallest $\Phi_{i}$ value is positioned last in the ordering.

## Results and discussion

### Experiment results

#### Effect of cement dust and barium sulfate combinations on friction and wear performance

**Figure 3:**
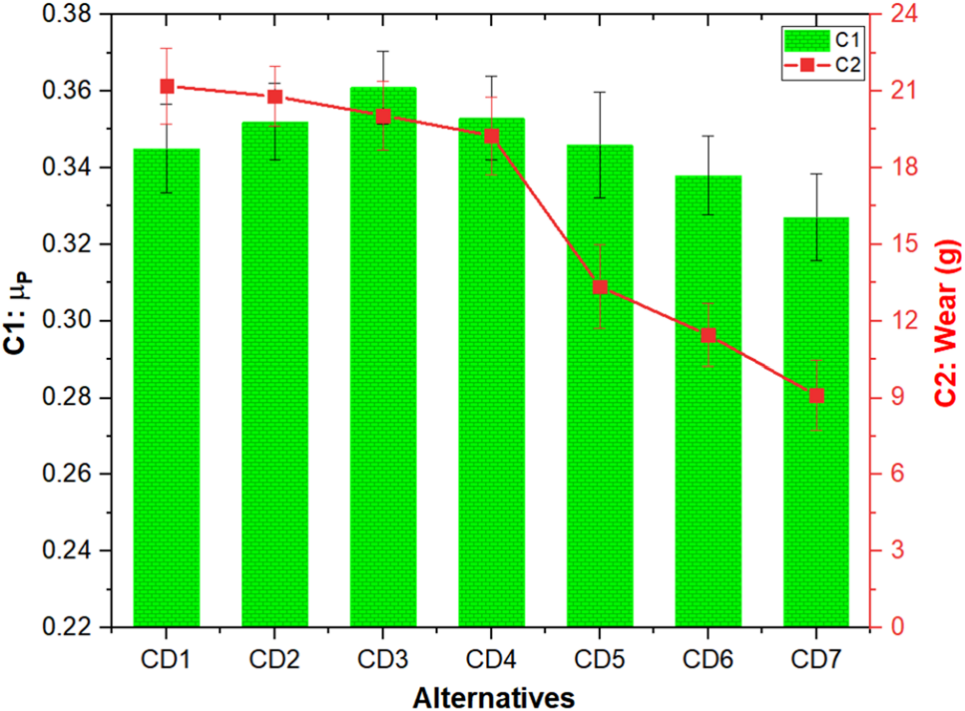
C1 (µ_P_) and C2 (wear) response of the composites.

The influence of cement dust and barium sulfate combination on the C1 (µ_P_) and C2 (wear) of the composites is presented in Fig. 3. The µ_P_ value remains 0.345 for CDI composite having 50 wt.% cement dust. Incorporating barium sulfate up to 20 wt% in the composite led to an increment of ~ 5% in μ_P_. After attaining a peak (0.361) for CD3 composites, the µ_P_ decreased with the further addition of barium sulfate. The lowest µ_P_ value of 0.327 was recorded for CD7 composites having 50 wt% barium sulfate. The wear remains highest (21.20 g) for 50 wt% cement dust, i.e., CD1 composites, decreased with increasing barium sulfate and decreasing cement dust contents. The lowest wear of 9.10 g was recorded for the composite with 50 wt% barium sulfate, i.e., CD7. Comparing the wear of composites CD1 and CD7 showed that composite CD7 was nearly 57% lower than composite CD1. High cement dust composites have a higher µ_P_ value due to inadequate bonding between the filler and resin, which causes increased wear, frictional fluctuation and friction-driven disc temperature rise. Furthermore, higher barium sulfate content aids in creating a load-carrying friction film, lowering µ_P_ and boosting wear resistance^45^. The observed trend of µ_P_ and wear are in good agreement with the literature. Composites' friction coefficient and wear resistance were found to rise with increasing slag waste and flyash content, as reported by Rajan et al.^46^ and Öztürk and Mutlu^17^. Dadkar et al.^13^, who investigated the effects of flyash and phenolic resin combinations, reported that composites loaded with greater flyash loadings exhibited enhanced µ_P_ and a commensurate drop in wear resistance. For red mud, waste slate powder, and slag waste-filled friction composites, Manoharan et al.^47^, Binda et al.^21^, and Wang et al.^16^ observed a similar tendency for friction and wear performance. In^23^, authors reported the friction performance and wear of the composites to increase linearly with increasing slag waste doses.

**Figure 4:**
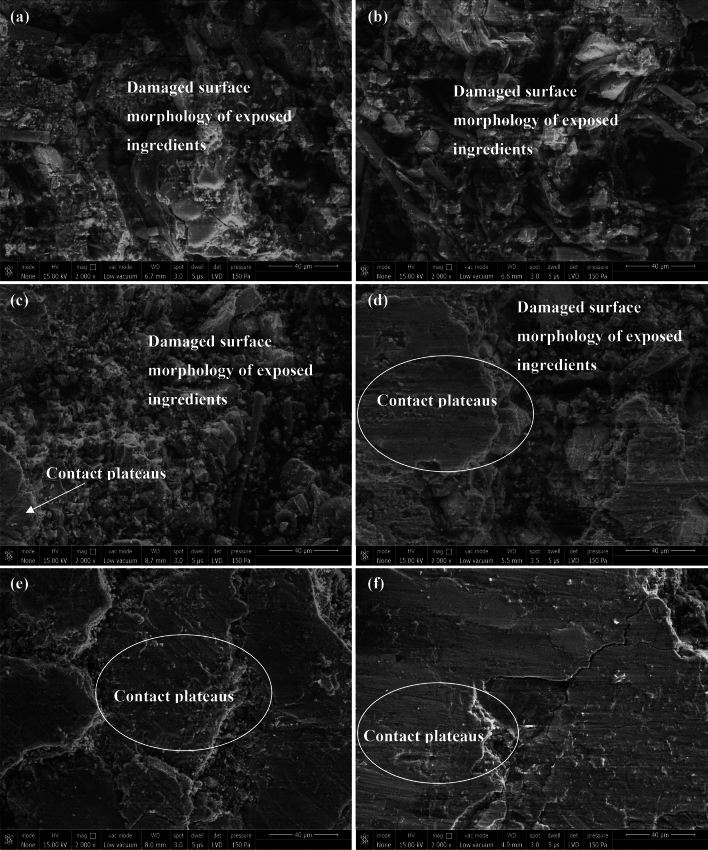
SEM micrographs of friction composites (**a**) CD1, (**b**) CD2, (**c**) CD3, (**d**) CD5, (**e**) CD6, and (**f**) CD7.

Realizing composites' friction and wear behavior may be significantly aided by gaining insight into the shape of the worn surfaces. Scanning electron micrographs of the worn composite surfaces were taken using a Zeiss SUPRA 40 VP microscope and are shown in Fig. 4. It is widely established that the morphological characteristics, such as hard particles, wear debris, contact/friction film, play an essential role in determining the tribological properties of friction materials^48–50^. When a brake is applied, the composite and the revolving disc come into physical contact, creating wear debris that aids in developing contact/friction film^11,51,52^.

**Figure 5:**
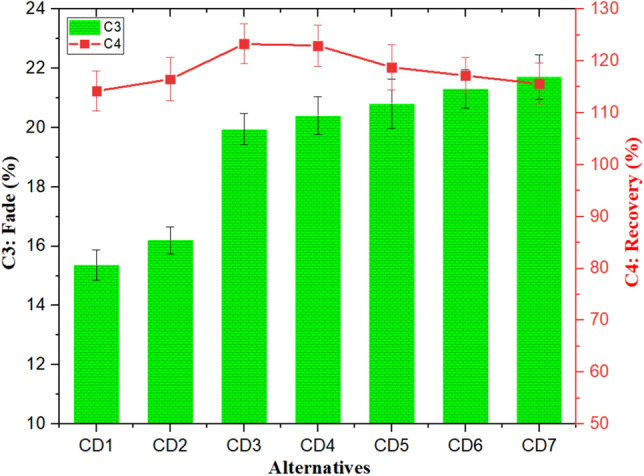
Results of C3 (fade (%)) and C4 (recovery (%)).

An ideal friction/contact film is coherent, thin, and uniform for good friction materials. A good contact/friction film will prevent the composite components from being easily worn away by sliding. Additionally, the development of a coherent, thin, and homogenous friction/contact film contributes to better friction stability in addition to reduced wear^51^. As shown in Fig. 4a and b, samples CD1 and CD2's worn surfaces were seriously damaged and had a rougher appearance than the other studied composites. The fact that no identifiable friction/contact film formed in CD1 or CD2 further proves that composite surfaces do not promote the compaction of debris to form friction/contact film but rather experience wear via thermo-mechanical deterioration. In contrast, CD3 (Fig. 4c) exhibits characteristics that indicate the lack of friction/contact film development, fiber debonding, and scattered wear debris. Increased wear is the ultimate result of such surface features, as the debris that fills the dislodged cavities tends to come apart due to a lack of sufficient compression to form a friction/contact film. A conclusion that can be drawn from the wear study is that wear resistance increases with increasing barium sulfate concentration. This increase could be attributed to friction/contact film forming on composites (CD5/CD6/CD7) containing a higher barium sulfate concentration (Fig. 4d–f). For instance, the composite CD5 (Fig. 5d) reveals relatively uniform topographic features and compacted wear debris. As seen in the micrographs of Fig. 4e and f, the area of the developed friction/contact film grows with increasing amounts of barium sulfate. Experimental results showed that composite CD7 (Fig. 4f), which included the most barium sulfate, exhibited the least amount of wear due to its more substantial potential to build a friction/contact layer without significant particle dislodging and fiber breakage.

#### Effect of cement dust and barium sulfate combinations on fade-recovery performance

The influence of cement dust and barium sulfate combinations on C3 (fade (%)) and C4 (recovery (%)) is presented in Fig. 5. The fade (%) remained lowest (15.36%) for CD1 composite and increased with increased barium sulfate content. Composite CD7 had 50 wt% barium sulfate has shown the highest fade (%) of 21.71% among all the fabricated composites. The high fade (%) response of barium sulfate-based composites is due to producing a frictional layer/film, which increases the interfacial contact area and reduces applied pressure, leading to fade^13,53^. The literature and the observed decreasing trend in fade (%) with increasing waste cement dust are in excellent accord. Similar results for decreased fade (%) with increased slag waste and flyash were reported by Rajan et al.^53^ and Dadkar et al.^13^, respectively. It is noteworthy here that the fade (%) of the investigated composites has been in the acceptable range of 0–30% as per IS-2742 standard.

On the other hand, recovery (%) fluctuated between 114.20 and 123.27% and remained in the acceptable range of 90–140% as per the IS-2742 standard. The recovery (%) remains lowest (114.20%) for CD1, having 50 wt.% cement dust content, after that for cement dust-to-barium sulfate proportion of 30:20, i.e., CD3, it improves to 123.27% and remains highest. The further addition of barium sulfate (≥ 25 wt%) deteriorates it to 115.60%. The higher recovery (%) was more toward the barium sulfate-dominated response. Incorporating barium sulfate, which promotes the rapid formation-deformation-reformation dynamics of the operating friction layer at the braking interface, may account for the increased recovery (%)^45^.

#### Effect of cement dust and barium sulfate combinations on stability and variability coefficient

**Figure 6:**
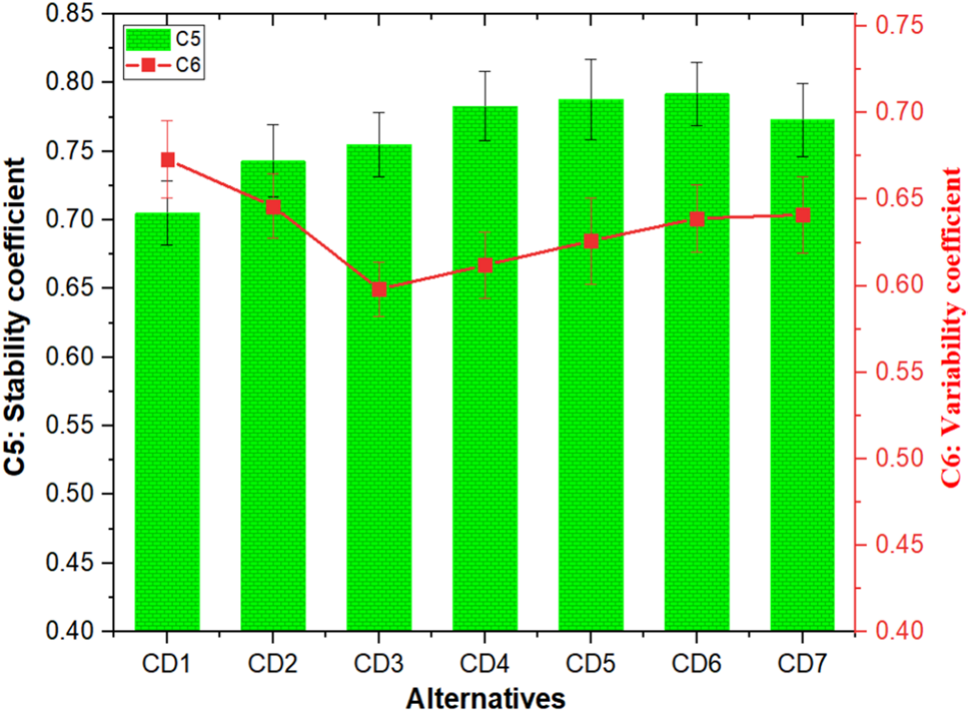
C5 (stability coefficient) and C6 (variability coefficient) response of the composites.

The influence of cement dust and barium sulfate combinations on C5 (stability coefficient) and C6 (variability coefficient) is depicted in Fig. 6. The stability coefficient remains lowest (0.705) for CD1 composite and increases with increased barium sulfate. The stability coefficient remains highest (0.792) for CD6 composite having 40 wt% barium sulfate content. This increase in composites' stability coefficient was credited to creation of load-carrying friction film with increased barium sulfate content^45^.

As seen in Fig. 6, there was a decrease in the variability coefficient of the composites upon decreasing the amount of cement dust from 50 to 30 wt% and increasing the amount of barium sulfate from 0 to 20 wt%. Then the variability coefficient of the composites increases below 30 wt% cement dust and above 20 wt% barium sulfate contents. It was observed that the CD3 composite shows the lowest variability coefficient of 0.598, whereas composite CD1 shows the highest variability coefficient of 0.673. Rajan et al.^46^ also observed a similar pattern in the stability coefficient while examining friction composites filled with slag waste. The researchers concluded that the stability coefficient exhibits an upward trend as the loading of slag waste rises in friction composites. Furthermore, the stability coefficient reaches its peak value when 60 wt.% of slag waste is added. However, it subsequently decreases with the addition of further slag waste. In their research on the effects of combining flyash and glass fiber, Jaggi et al.^54^ observed that as the flyash loadings in the composites rose, there was a rise in the variability coefficient and a corresponding drop in the stability coefficient.

#### Effect of cement dust and barium sulfate combinations on friction fluctuations and disc temperature rise

**Figure 7:**
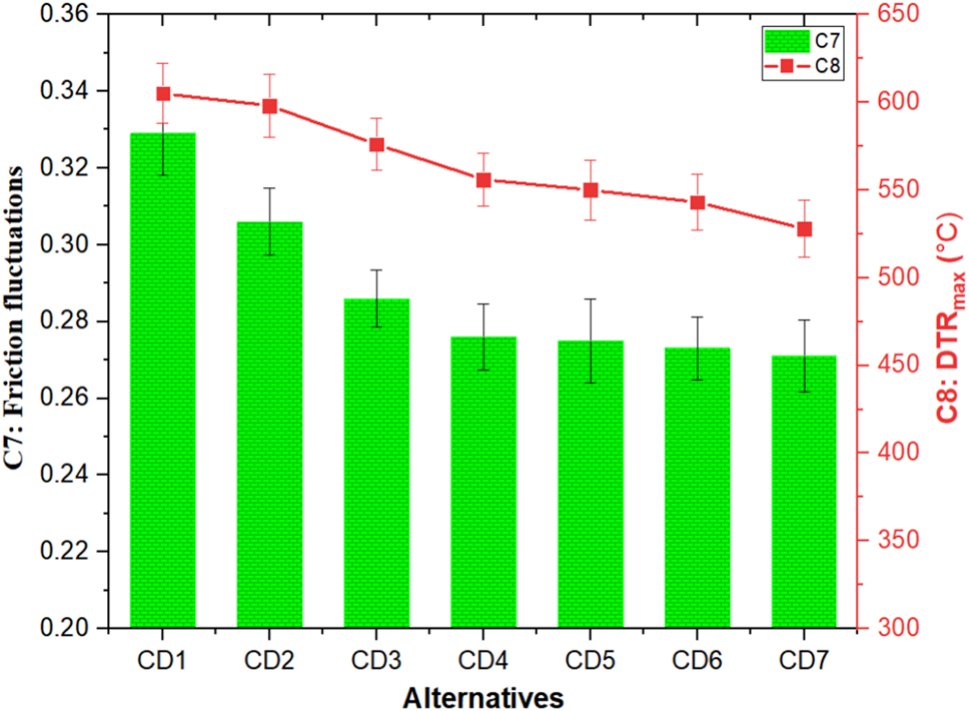
C7 (frictional fluctuations) and C8 (DTR_max_) response of the composites.

The influence of cement dust and barium sulfate combinations on C7 (frictional fluctuations) and C8 (DTR_max_) is depicted in Fig. 7. It was observed from Fig. 7 that frictional fluctuations and DTR_max_ showed a decreasing tendency with the decrease in cement dust with a simultaneous increase in barium sulfate content. The friction fluctuations remained highest (0.329) for CD1 composite with 50 wt% cement dust, reduced by ~ 18% and remained lowest (0.271) for 50 wt% barium sulfate added composite, i.e., CD7. Using a comparative approach, Tiwari et al.^45^ evaluated the tribological performance of brake friction composites based on barium sulfate and cenosphere. The author posited that the friction fluctuations in composites filled with 60 wt.% cenospheres were consistently greater than those observed in composites loaded with 60 wt.% barium sulfate. Jaggi et al.^54^ also observed heightened friction fluctuations with higher flyash content. Further, it was observed from Fig. 7 that DTR_max_ decreased continuously with the decrease in cement dust and simultaneous increase in barium sulfate content. The CD1 composite was found to have the greatest DTR_max_ value (605 °C). Similar findings were reported by Rajan et al.^53^; authors reported that DTR_max_ remained highest for composites containing 60 wt.% flyash and decreased with decreasing filler loading.

**Figure 8:**
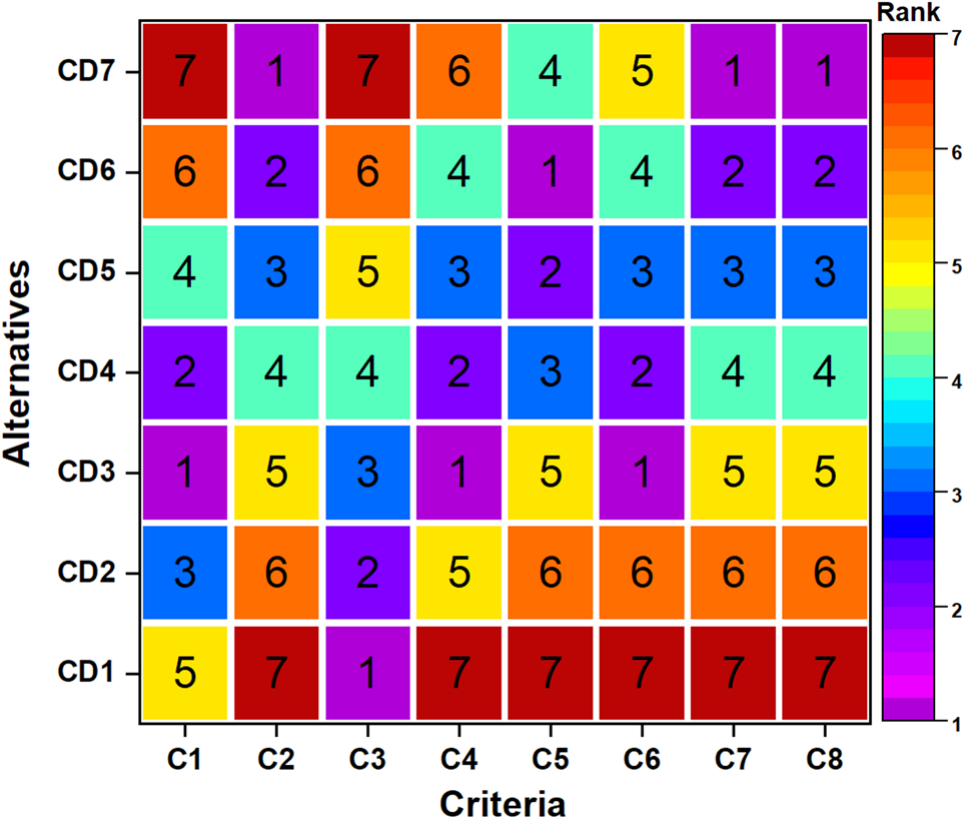
Alternatives ranking based on individual criteria.

The initial decrease in CD2 on incorporating 10 wt% of barium sulfate was by 7 °C followed by a drop of 29 °C for CD3 and a drop of 62 °C for CD6 at 20 and 40 wt% of barium sulfate, respectively. Whereas for 50 wt% barium sulfate added composite (i.e. CD7) the DTR_max_ decreased by 77 °C. A consistent decline in DTR_max_ can be explained by improved heat dissipation at the braking interface. Composite CD7 had the lowest DTR_max_ value (i.e., 528 °C) and was more towards a barium sulfate-dominated response. According to Satapathy et al.^13,55^, in their investigations on the effect of flyash with phenolic resin and vermiculite, it was observed that the DTR_max_ of composites displayed an upward trend with increasing flyash content. The evaluated automotive brake friction composite alternatives can be ranked according to their efficacy for each criterion identified in Fig. 8.

The results demonstrate that no composite alternative performed best using all performance metrics. For example, composite alternative CD3 exhibited the highest µ_P_ (0.361) and recovery (123.27%). The composite alternative CD7 indicates the most significant performance of wear (9.1 g), friction fluctuations (0.271), and DTR_max_ (528 °C), but it exhibits the lowest performance for µ_P_ (2.56 GPa), fade (21.71%), and next-to-lowest recovery (%). The composite alternative CD1 indicates the highest fade performance (15.36%) but displays the lowest performance for other criteria except for µ_P_. Moreover, the composite alternative CD6 shows the highest stability coefficient (0.792) and second-best wear performance (11.45 g) and DTR_max_ (543 °C). It becomes challenging to select the optimal composite alternative by taking all criteria. Therefore, to pick the best composite alternative by considering all requirements simultaneously, a hybrid AHP-MABAC methodology is used.

### Optimal composite alternative selection

The ranking results are presented in three sections. First, the AHP method's results for determining the criteria weights are discussed. Second, the AHP method outputs are considered in the MABAC calculation for the final ranking. Third, a sensitivity analysis was carried out to assess the robustness of the proposed MCDM approach.

#### AHP results

The criterion weights have great importance in ranking analysis. At present AHP method is used for the estimation of criteria weight. A pairwise comparison matrix was structured using a nine-point scale, as presented in Table 4. The value "1" in the comparison matrix shows that the compared criteria are equally preferred. Furthermore, if the comparison between C1 and C2 receives, say, "1.50", specifying that C1 is 1.50 times more important than C2 and the comparison between C2 and C1 automatically receives the reciprocal of 1.50, i.e., 0.67.

**Table 4:** The comparison matrix.

	$C1$	$C2$	$C3$	$C4$	$C5$	$C6$	$C7$	$C8$
$C1$	1.00	1.50	1.00	1.33	1.75	2.00	3.00	4.00
$C2$	0.67	1.00	0.90	0.80	1.25	1.50	2.00	3.00
$C3$	1.00	1.11	1.00	1.10	1.50	1.75	2.25	3.25
$C4$	0.75	1.25	0.91	1.00	1.40	1.60	2.00	3.00
$C5$	0.57	0.80	0.67	0.71	1.00	1.25	2.20	3.25
$C6$	0.50	0.67	0.57	0.63	0.80	1.00	1.75	1.20
$C7$	0.33	0.50	0.44	0.50	0.45	0.57	1.00	1.25
$C8$	0.25	0.33	0.31	0.33	0.31	0.83	0.80	1.00

The computed criteria weights are listed in Table 5. The order of criteria weight was obtained as C1 (0.1989) > C3 (0.1696) > C4 (0.1551) > C2 (0.1412) > C5 (0. 0.1242) > C6 (0.0935) > C7 (0.0658) > C8 (0.0517). Furthermore, the consistency test parameters were estimated using Eq. (5). The results are shown in Table 5. The generated CR value (0.0063) is less than the highest consistency limit of 10%, suggesting that the calculated criteria weights are appropriate for the ranking analysis.

**Table 5:** AHP results.

	Criteria							
	$C1$	$C2$	$C3$	$C4$	$C5$	$C6$	$C7$	$C8$
$\omega_{j}$	0.1989	0.1412	0.1696	0.1551	0.1242	0.0935	0.0658	0.0517
Consistency parameters	λ_max_ = 8.0617		RI = 1.404		CI = 0.0088		CR = 0.0063	

#### MABAC results

After defining the weights of the selected criteria, the AHP-MABAC technique was used to rank alternatives based on their overall performance. The structured decision matrix shown in Table 6 serves as the basis for the MABAC technique. The decision matrix was first normalized using Eqs. (6–7) in accordance with the MABAC technique, and the results are shown in Table 7.

**Table 6:** Decision matrix.

Alternatives	$C1$	$C2$	$C3$	$C4$	$C5$	$C6$	$C7$	$C8$
CD1	0.345	21.20	15.36	114.20	0.705	0.673	0.329	605
CD2	0.352	20.80	16.19	116.48	0.743	0.646	0.306	598
CD3	0.361	20.05	19.94	123.27	0.755	0.598	0.286	576
CD4	0.353	19.25	20.40	122.95	0.783	0.612	0.276	556
CD5	0.346	13.35	20.81	118.78	0.788	0.626	0.275	550
CD6	0.338	11.45	21.30	117.16	0.792	0.639	0.273	543
CD7	0.327	09.10	21.71	115.60	0.773	0.641	0.271	528

**Table 7:** Decision matrix normalization.

	$C1$	$C2$	$C3$	$C4$	$C5$	$C6$	$C7$	$C8$
CD1	0.5294	0.0000	1.0000	0.0000	0.0000	0.0000	0.0000	0.0000
CD2	0.7353	0.0331	0.8693	0.2514	0.4368	0.3600	0.3966	0.0909
CD3	1.0000	0.0950	0.2787	1.0000	0.5747	1.0000	0.7414	0.3766
CD4	0.7647	0.1612	0.2063	0.9647	0.8966	0.8133	0.9138	0.6364
CD5	0.5588	0.6488	0.1417	0.5050	0.9540	0.6267	0.9310	0.7143
CD6	0.3235	0.8058	0.0646	0.3264	1.0000	0.4533	0.9655	0.8052
CD7	0.0000	1.0000	0.0000	0.1544	0.7816	0.4267	1.0000	1.0000

Following that, Eq. (8) was used to compute the weighted normalized decision matrix. The weighted normalized values of the first criteria, for example, are determined as follows:

$$\begin{array}{*{20}l} {\Re_{11}^{ * } = \omega_{1} + (\Re_{11} \times \omega_{1} ) = {0}{\text{.1989}} + (0.5294 \times {0}{\text{.1989}}) = 0.3042} \hfill \\ {\Re_{21}^{ * } = \omega_{1} + (\Re_{21} \times \omega_{1} ) = {0}{\text{.1989}} + ({0}{\text{.7353}} \times {0}{\text{.1989}}) = 0.3452} \hfill \\ {\Re_{31}^{ * } = \omega_{1} + (\Re_{31} \times \omega_{1} ) = {0}{\text{.1989}} + ({1}.0000 \times {0}{\text{.1989}}) = 0.3978} \hfill \\ \vdots \hfill \\ {\Re_{71}^{ * } = \omega_{1} + (\Re_{71} \times \omega_{1} ) = {0}{\text{.1989}} + (0 \times {0}{\text{.1989}}) = 0.1989} \hfill \\ \end{array}$$

The other values of the criteria are determined accordingly and the formulated weighted normalized decision matrix is presented in Table 8.

**Table 8:** Weighted normalized decision matrix.

	$C1$	$C2$	$C3$	$C4$	$C5$	$C6$	$C7$	$C8$
CD1	0.3042	0.1412	0.3392	0.1551	0.1242	0.0935	0.0658	0.0517
CD2	0.3452	0.1459	0.3170	0.1941	0.1784	0.1272	0.0919	0.0564
CD3	0.3978	0.1546	0.2169	0.3102	0.1956	0.1870	0.1146	0.0712
CD4	0.3510	0.1640	0.2046	0.3047	0.2356	0.1695	0.1259	0.0846
CD5	0.3101	0.2328	0.1936	0.2334	0.2427	0.1521	0.1271	0.0886
CD6	0.2633	0.2550	0.1806	0.2057	0.2484	0.1359	0.1293	0.0933
CD7	0.1989	0.2824	0.1696	0.1790	0.2213	0.1334	0.1316	0.1034

After weighted normalization, border approximation area matrix was structured using Eq. (9) and presented in Table 9. For example, the first criterion (C1) value is determined as follows:

$$\eta_{1} = \left( {\Re_{11}^{ * } \times \Re_{21}^{ * } \times \Re_{31}^{ * } \times \Re_{41}^{ * } \times \Re_{51}^{ * } \times \Re_{61}^{ * } \times \Re_{71}^{ * } } \right)^{1/7}$$

$$\eta_{1} = \left( {0.3042 \times 0.3452 \times 0.3978 \times 0.3510 \times 0.3101 \times 0.2633 \times 0.1989} \right)^{1/7}$$

$$\eta_{1} = \left( {0.000238} \right)^{1/7} = 0.3036$$

**Table 9:** Border approximation area matrix.

	$C1$	$C2$	$C3$	$C4$	$C5$	$C6$	$C7$	$C8$
$\eta_{j}$	0.3036	0.1894	0.2240	0.2194	0.2018	0.1397	0.1095	0.0763

Next, the distance of weighted normalized decision matrix elements (Table 8) from formulated border approximation area matrix (Table 9) is determined using Eq. (10) and listed in Table 10 as follows:

$$\begin{array}{*{20}l} {\delta_{11} = \Re_{11}^{ * } - \eta_{1} = 0.3042 - 0.3036 = 0.0006} \hfill \\ {\delta_{21} = \Re_{21}^{ * } - \eta_{1} = 0.3452 - 0.3036 = 0.0415} \hfill \\ {\delta_{31} = \Re_{31}^{ * } - \eta_{1} = 0.3978 - 0.3036 = 0.0942} \hfill \\ \vdots \hfill \\ {\delta_{71} = \Re_{71}^{ * } - \eta_{1} = 0.1989 - 0.3036 = - 0.1047} \hfill \\ \end{array}$$

**Table 10:** The alternative's distance from the formulated border approximation area matrix.

	$C1$	$C2$	$C3$	$C4$	$C5$	$C6$	$C7$	$C8$
CD1	0.0006	− 0.0482	0.1152	− 0.0643	− 0.0776	− 0.0462	− 0.0437	− 0.0246
CD2	0.0415	− 0.0436	0.0930	− 0.0253	− 0.0233	− 0.0126	− 0.0176	− 0.0199
CD3	0.0942	− 0.0348	− 0.0071	0.0908	− 0.0062	0.0473	0.0051	− 0.0051
CD4	0.0474	− 0.0255	− 0.0194	0.0853	0.0338	0.0298	0.0164	0.0083
CD5	0.0064	0.0434	− 0.0304	0.0140	0.0409	0.0124	0.0176	0.0124
CD6	− 0.0404	0.0655	− 0.0434	− 0.0137	0.0466	− 0.0038	0.0198	0.0171
CD7	− 0.1047	0.0930	− 0.0544	− 0.0404	0.0195	− 0.0063	0.0221	0.0271

Finally, using Eq. (11), the composite score index ($\Phi_{i}$) was calculated for each composite option and is shown in Table 11. It is observed that the composite alternative CD3 has the highest value (0.1841), followed by CD4 (0.1762), and the composite alternative CD1 has the lowest value (− 0.1888). Based on this analysis, it was found that the formulation CD3, containing 30 wt% cement dust and 20 wt% barium sulfate performed best.

**Table 11:** Overall score index and alternatives ranking using AHP-MABAC approach.

	Alternatives						
	CD1	CD2	CD3	CD4	CD5	CD6	CD7
$\Phi_{i}$	− 0.1888	− 0.0077	0.1841	0.1762	0.1167	0.0477	− 0.0441
Rank	7	5	1	2	3	4	6

#### Sensitivity analysis

##### Sensitivity analysis using variation in criteria weight

**Figure 9:**
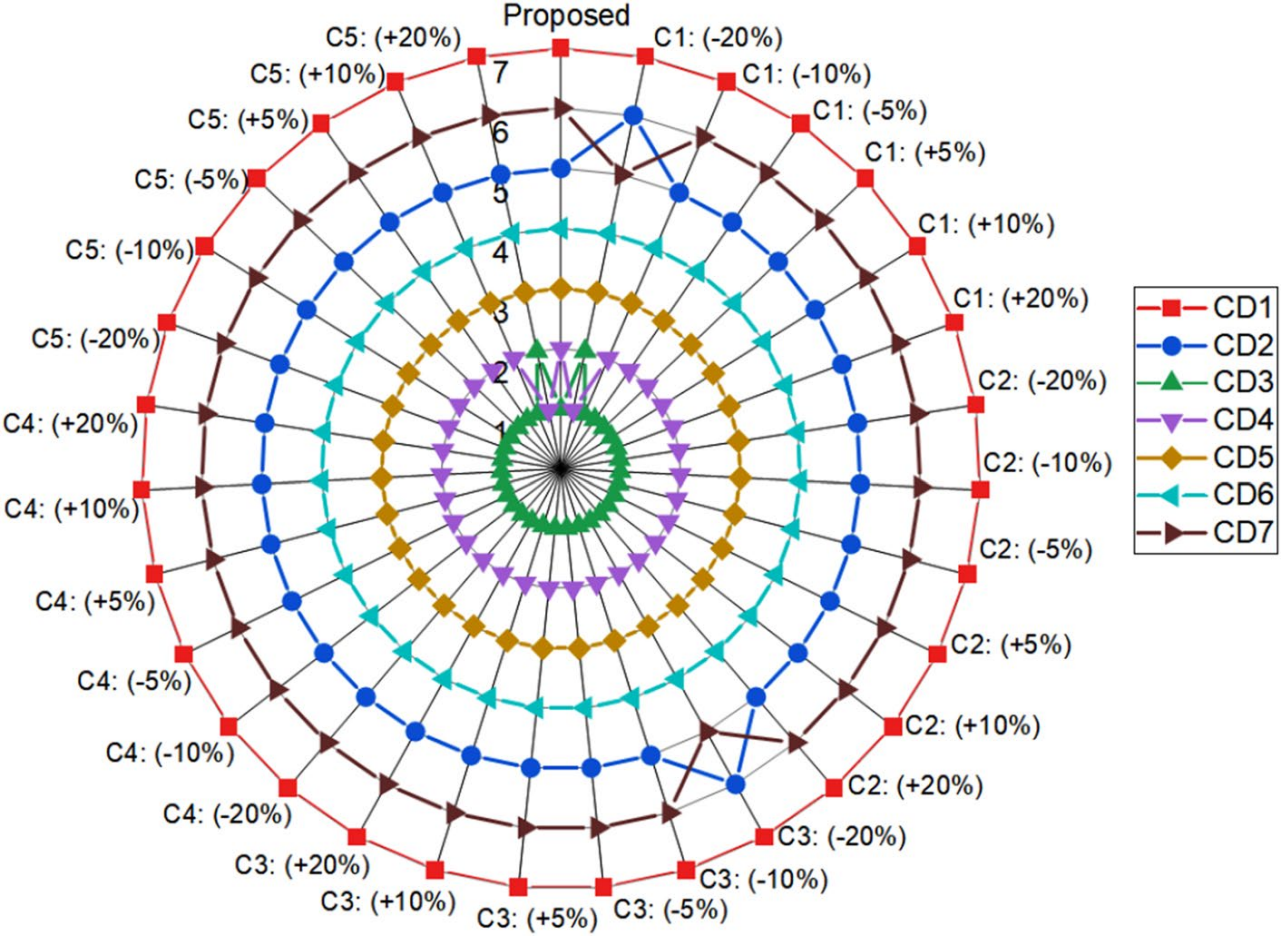
Sensitivity analysis.

To further examine the ranking's consistency and reliability with respect to the weight of the criteria, a sensitivity analysis was also conducted. The μ_P_ (C1), wear (C2), fade (C3), recovery (C4), and stability coefficient (C5) are the five most weighted factors for sensitivity analysis, with respective weights of 0.1989, 0.1412, 0.1696, 0.1551 and 0.1242. In order to perform the sensitivity analysis, a criterion weight was increased and decreased in three stages (5%, 10%, and 20%). To maintain the overall weight of all criteria at one, the weights of the remaining seven criteria have been proportionally changed. Figure 9 depicts how the ordering of the composite alternative changes when the weights of the selected criteria are altered for the purpose of sensitivity analysis.

Based on the data in Fig. 9, all composite alternatives retain their respective rankings when the weights of the selected criterion are increased and decreased in two steps of 5% and 10%. In these scenarios, the ranking order is recorded as CD3 > CD4 > CD5 > CD6 > CD2 > CD6 > CD7. For C1 (− 20%), C3 (− 20%), and C5 (+ 20%), a minor change in the ranking order of the composite alternatives was recorded. A little sensitivity in the composite alternative ranking is observed where first-ranked, second-ranked (CD3 and CD4), and fifth-ranked and sixth-ranked alternatives swap places (CD2 and CD7).

##### Sensitivity analysis using objective weighting approach

The ranking of the composites was further analyzed using CRITIC, an objective weighting approach. According to existing research, CRITIC has emerged as the dominant objective approach for criteria weighting. The main reason for CRITIC's popularity is its consideration of the contrasting intensities and contradictory connections created by each selected criterion in the decision-making problem. The main steps involved in the CRITIC approach are decision matrix normalization, standard deviation estimation for each criterion, building a correlation coefficient between criteria, and information measures for each criterion^29^. The weight of different criteria using CRITIC methods are C1 (0.1318), C2 (0.1494), C3 (0.2362), C4 (0.1073), C5 (0.0906), C6 (0.0816), C7 (0.0933), and C8 (0.1098). Using the criterion weight determined with the CRITIC method, the MABAC analysis was performed to obtain the ranks for the alternatives, and the results are presented in Table 12.

**Table 12:** Overall score index and alternatives ranking using CRITIC-MABAC approach.

	Alternatives						
	CD1	CD2	CD3	CD4	CD5	CD6	CD7
$\Phi_{i}$	− 0.1458	− 0.0017	0.1116	0.1281	0.1094	0.0676	0.0230
Rank	7	6	2	1	3	4	5

The ranks of the composite alternatives by the CRITIC-MABAC method presented in Table 12 recommended alternative CD4 as it is the first-ranked. In contrast, composite alternative CD3 was the first-ranked (Table 11) according to the AHP-MABAC method. Also, the fifth- and sixth-ranked alternatives noticed a swap in ranking for changing criterion weight from AHP to CRITIC. Finally, CD4 is the worst alternative since it ranks last according to AHP-MABAC and CRITIC-MABAC methods. In order to determine the statistical significance of the difference between the ranks obtained by the AHP-MABAC and CRITIC-MABAC approaches, the Spearman correlation coefficient [Eq. (12)] was used^56^.

12 $${\text{Spearman}}\;{\text{correlation}}\;{\text{coefficient }} = 1 - \frac{{6\sum\nolimits_{i = 1}^{p} {{\rm Y}_{i}^{2} } }}{{p^{3} - p}}$$

where, the number of composite alternatives is $p$, while $\Upsilon_{i}$ represents the rank difference for the i-th alternative in AHP-MABAC and CRITIC-MABAC weighting conditions.

The Spearman correlation coefficient value between the AHP-MABAC and CRITIC-MABAC methods was greater than 0.92, suggesting a very high correlation. The correlation coefficient lets us conclude that the obtained rank is credible.

#### Validation using other MCDM techniques

One of the most important questions to be answered before making a final decision is whether the proposed AHP-MABAC approach generates background data. To answer this, the ranking results obtained with the proposed approach were compared with other decision-making models for its effective implementation. Using the criterion weight determined with the AHP method [Eq. (4)], MCDM methods including GRA^15^, PROMETHEE^30^, and VIKOR^31^ were used to rank the composite alternatives. The mathematical form of these methods involved various steps as follows.

##### GRA method

Step 1: After decision matrix normalization using Eqs. (6) and (7), a difference matrix was structured as;

13 $$\hbar_{ij} = \left| {1 - \max_{i = 1}^{p} \left\{ {\Re_{ij} } \right\}} \right|$$

Step-2: Calculation of grey correlation coefficient using following formula:

14 $$\psi_{ij} = \frac{{\min_{i = 1}^{p} \min_{i = 1}^{q} \hbar_{ij} + 0.5 \times \max_{i = 1}^{p} \max_{i = 1}^{q} \hbar_{ij} }}{{\hbar_{ij} + 0.5 \times \max_{i = 1}^{p} \max_{i = 1}^{q} \zeta_{ij} }}$$

Step-3: Finally, grey correlation grading (GRG) is calculated for each criterion using Eq. (15).

15 $${\text{GRG }} = \frac{1}{q}\sum\limits_{j = 1}^{q} {\left[ {\omega_{j} \times \psi_{ij} } \right]}$$

Following the GRG value, the alternatives are then ordered in decreasing order. The alternative at the top of the list is the most favoured.

##### PROMETHEE method

Step 1: After decision matrix normalization using Eqs. (6) and (7), a preference function $\kappa_{j} (i,i^{\prime})$ is calculated as;

16 $$\kappa_{j} (i,i^{\prime}) = 0\quad {\text{if}}\;\Re_{ij} \le \Re_{{i^{\prime}j}} ,\;{\text{and}}\;\;\kappa_{j} (i,i^{\prime}) = (\Re_{ij} - \Re_{{i^{\prime}j}} )\quad {\text{if}}\;\Re_{ij} { > }\Re_{{i^{\prime}j}}$$

Step 2: Calculation of weighted preference function using following formula:

17 $$\varpi (i,i^{\prime}) = \sum\limits_{i = 1}^{p} {\omega_{j} \times \kappa_{j} (i,i^{\prime})}$$

Step 3: The positive and negative outranking flows are determined as:

18 $$\chi_{i}^{ + } = \frac{1}{p - 1}\sum\limits_{{i^{\prime} = 1}}^{p} {\varpi (i,i^{\prime})} ,{\text{ and}}$$

19 $$\chi_{i}^{ - } = \frac{1}{p - 1}\sum\limits_{{i{\prime} = 1}}^{p} {\varpi (i^{\prime},i)} \,$$

Step 4: Finally, the net outranking flow for each alternative is determined as:

20 $${\text{Net}}\;{\text{outranking}}\;{\text{flow }} = \chi_{i}^{ + } - \chi_{i}^{ - }$$

The alternatives are then arranged in descending order according to the value of their net outranking flow. The alternative at the top of the list is the most preferred one.

##### VIKOR method

Step 1: After structuring decision matrix, the utility measure (UM_i_) and regret measure (RM_i_) are determined using following equations;

21 $${\text{UM}}_{{\text{i}}} = \sum\limits_{j = 1}^{q} {\frac{{\omega_{j} \left[ {\Gamma_{i}^{ + } - \Gamma_{ij} } \right]}}{{\Gamma_{i}^{ + } - \Gamma_{i}^{ - } }}} ,\;\;\;\;\;\;\;{\text{if}}\;{\text{ j}}\; \in \;{\text{preferable }}\;{\text{criteria}}$$

22 $${\text{UM}}_{{\text{i}}} = \sum\limits_{j = 1}^{q} {\frac{{\omega_{j} \left[ {\Gamma_{ij} - \Gamma_{i}^{ - } } \right]}}{{\Gamma_{i}^{ + } - \Gamma_{i}^{ - } }}} ,\;\;\;\;\;\;{\text{if}}\;{\text{ j}}\; \in \;{\text{non - preferable}}\;{\text{ criteria}}$$

23 $${\text{RM}}_{{\text{i}}} = Max^{x} \;of\;\left\{ {\frac{{\omega_{j} \left[ {\Gamma_{i}^{ + } - \Gamma_{ij} } \right]}}{{\Gamma_{i}^{ + } - \Gamma_{i}^{ - } }}\;} \right\}$$

*Step V:* VIKOR index ($\Omega_{i}$) is calculated as:

24 $${\text{VIKOR}}\;{\text{ index }} = (0.5)\left( {\frac{{\left( {UM_{i} - UM_{i}^{ - } } \right)}}{{\left( {UM_{i}^{ + } - UM_{i}^{ - } } \right)}}} \right) + \left( {0.5} \right)\left( {\frac{{\left( {RM_{i} - RM_{i}^{ - } } \right)}}{{\left( {RM_{i}^{ + } - RM_{i}^{ - } } \right)}}} \right)$$

where, $$UM_{i}^{ + } = \max \left[ {UM_{i} ,\;i = 1,\;2...p} \right]$$; $$UM_{i}^{ - } = \min \left[ {UM_{i} ,\;i = 1,\;2...p} \right]$$.

$$RM_{i}^{ + } = \max \left[ {RM_{i} ,\;i = 1,\;2...p} \right]$$; $$RM_{i}^{ - } = \min \left[ {RM_{i} ,\;i = 1,\;2...p} \right]$$.

The alternatives are then ranked according to their VIKOR index. The alternative with the lowest VIKOR index is the most preferred one.

**Figure 10:**
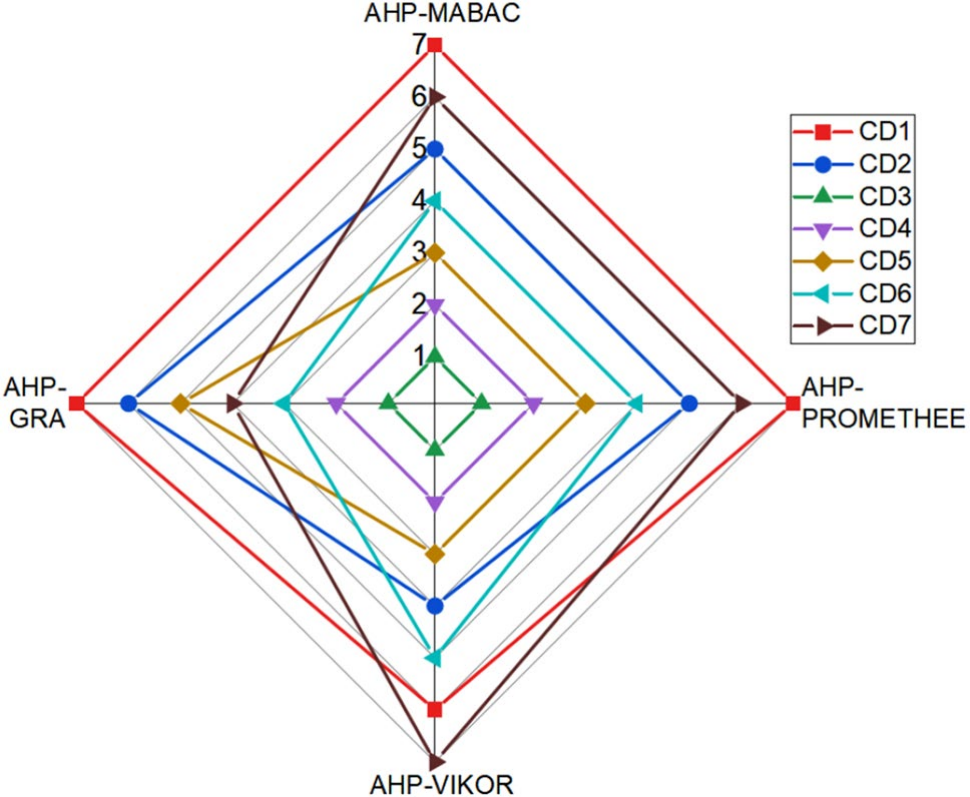
Alternatives ranking with respect to various models.

Figure 10 depicts a comparison of the AHP-MABAC approach's ranking results with those obtained by the AHP-GRA^15^, AHP-PROMETHEE^30^, and AHP-VIKOR^31^ models.

**Figure 11:**
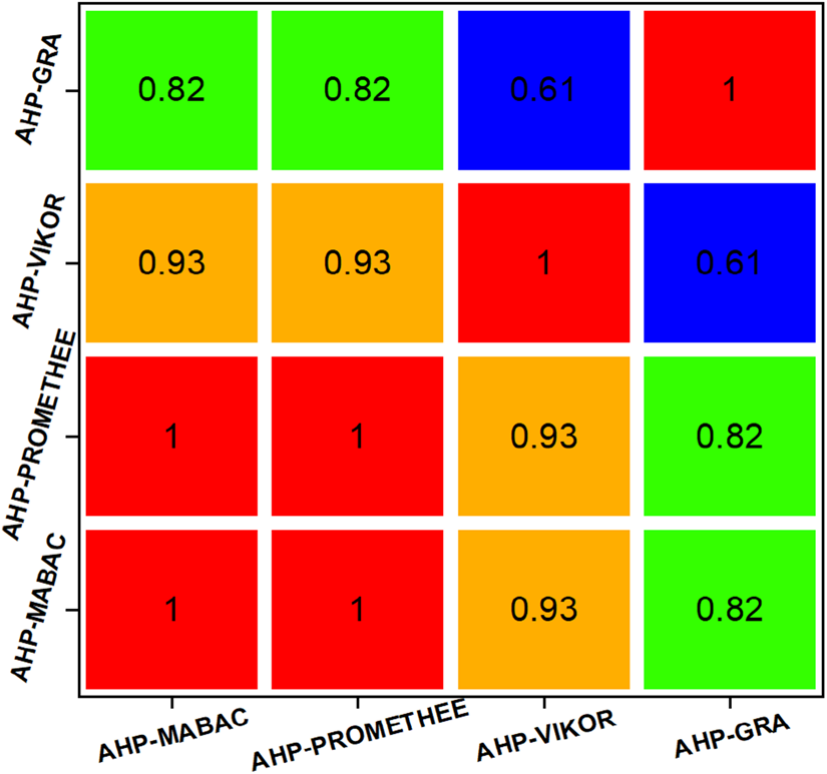
Spearman’s rank correlation coefficients.

The comparative ranking results in Fig. 10 demonstrate that the alternative CD3 is the most dominant, ranked first across all methods. Alternatives CD4 and CD5 are ranked second and third in all methods except AHP-GRA, where alternative CD5 replaces CD6. Finally, CD1 and CD7 are the worst options because they are ranked sixth and seventh in all models except AHP-GRA, where CD7 is ranked fourth. No single decision-making model gives an accurate ranking because the position of the options varies with the technique used. Consequently, the Spearman correlation coefficient (Eq. 12) was utilized to study the statistical significance of differences in ranks obtained by the AHP-MABAC approach and other models, as shown in Fig. 11. The results show a statistically significant correlation in the ranking of the comparative decision-making models. For AHP-MABAC, the correlation values are greater than 0.82, indicating that the proposed AHP-MABAC approach is highly correlated. A substantial correlation can be inferred from the fact that all of the correlation coefficients have a value that is greater than 0.61, with a mean value of 0.85. As a consequence of this, one can reach the conclusion that the suggested ranking is both accurate and credible.

## Conclusions

This research proposes a multi-criteria decision-making model to select friction composites with excellent tribological properties as automotive braking components. Seven composites containing varying proportions of cement dust (50 to 0 wt%) and barium sulfate (0 to 50 wt%) were fabricated and tribo-evaluated on the Krauss machine using European norms. It has been observed that the change in cement dust and barium sulfate amount has led to a substantial difference in the evaluated tribological properties. The friction coefficient and fade performance increased with increased cement dust content. In contrast, increased barium sulfate resulted in a composite with decreased frictional fluctuations, lower disc temperature rise, and enhanced wear resistance. A hybrid AHP-MABAC model was used to determine the final rankings because no single overall composite option could satisfy all performance requirements. Based on the results of the AHP-MABAC evaluation, the composites with a weight distribution of 30% cement dust and 20% barium sulfate displayed the most desirable tribological characteristics. Finally, the results were validated by performing a well-defined sensitivity analysis. The Spearman correlation coefficient-based results were thoroughly reviewed and compared to the effects of various decision-making models, namely AHP-PROMETHEE, AHP-VIKOR, and AHP-GRA. The results achieved by the AHP-MABAC method proved reliable, and the proposed approach can be used to solve other decision-making problems as an effective tool. In the future, studies can be conducted on life cycle analysis, brake squeal noise, and airborne particle emissions of automotive friction composite materials loaded with waste cement dust.

## Data Availability

The datasets used and/or analyzed during the current study available from the corresponding author on reasonable request.
